# Genetic variation for tolerance to high temperatures in a population of *Drosophila melanogaster*

**DOI:** 10.1002/ece3.4409

**Published:** 2018-10-11

**Authors:** Carmen Rolandi, John R. B. Lighton, Gerardo J. de la Vega, Pablo E. Schilman, Julián Mensch

**Affiliations:** ^1^ IBBEA‐CONICET‐UBA. DBBEA Facultad de Ciencias Exactas y Naturales Universidad de Buenos Aires Buenos Aires Argentina; ^2^ Sable Systems International Las Vegas Nevada; ^3^ Grupo de Ecología de Poblaciones de Insectos (GEPI) INTA EEA Bariloche Bariloche Argentina; ^4^ IEGEBA‐CONICET‐UBA DEGE Facultad de Ciencias Exactas y Naturales Universidad de Buenos Aires Buenos Aires Argentina

**Keywords:** climatic adaptation, CTmax, DGRP, global warming scenario, GWAS, SNPs

## Abstract

The range of thermal tolerance is one of the main factors influencing the geographic distribution of species. Climate change projections predict increases in average and extreme temperatures over the coming decades; hence, the ability of living beings to resist these changes will depend on physiological and adaptive responses. On an evolutionary scale, changes will occur as the result of selective pressures on individual heritable differences. In this work, we studied the genetic basis of tolerance to high temperatures in the fly *Drosophila melanogaster* and whether this species presents sufficient genetic variability to allow expansion of its upper thermo‐tolerance limit. To do so, we used adult flies derived from a natural population belonging to the *Drosophila* Genetic Reference Panel, for which genomic sequencing data are available. We characterized the phenotypic variation of the upper thermal limit in 34 lines by measuring knockdown temperature (i.e., critical thermal maximum [CTmax]) by exposing flies to a ramp of increasing temperature (0.25°C/min). Fourteen percent of the variation in CTmax is explained by the genetic variation across lines, without a significant sexual dimorphism. Through a genomewide association study, 12 single nucleotide polymorphisms associated with the CTmax were identified. In most of these SNPs, the less frequent allele increased the upper thermal limit suggesting that this population harbors raw genetic variation capable of expanding its heat tolerance. This potential upper thermal tolerance increase has implications under the global warming scenario. Past climatic records show a very low incidence of days above CTmax (10 days over 25 years); however, future climate scenarios predict 243 days with extreme high temperature above CTmax from 2045 to 2070. Thus, in the context of the future climate warming, rising temperatures might drive the evolution of heat tolerance in this population by increasing the frequency of the alleles associated with higher CTmax.

## 1 INTRODUCTION

The range of thermal tolerance is one of the main factors influencing the geographic distribution and abundance of species (Bozinovic, Calosi, & Spicer, 2011). Climate change projections predict increases in average and extreme temperatures over the coming decades (Coumou & Rahmstorf, 2012; Easterling, 2000), challenging the capacity of organisms to cope with such strong selective pressures. Hence, their ability to avoid demographic reductions or even extinction will depend on the adaptive potential of their upper thermal limits. Climate warming temperature events exceeding a species’ range of thermal tolerance that may therefore act as a driving force for evolution and species persistence (Parmesan, Root, & Willig, 2000). In particular, ectotherms constitute the majority of terrestrial organisms and because of their close association between environmental temperature and body temperature are likely to be negatively influenced by global warming (Deutsch et al., 2008). How ectotherms respond to rising temperatures will depend on a short‐term scale on the existence of thermoregulatory behavior and plastic changes of physiological limits (Sørensen, Kristensen, & Overgaard, 2016; Sunday et al., 2014). The critical thermal maximum (CTmax) is the temperature at which organisms lose motor control (Lutterschmidt & Hutchison, 1997) making them unable to escape temperature stress. Hence, if temperatures rise above that thermal threshold, behavioral thermoregulation might not buffer its impact. Furthermore, upper lethal temperature (ULT) lies very close to CTmax (Chown & Nicolson, 2004; de la Vega, Medone, Ceccarelli, Rabinovich, & Schilman, 2015; de la Vega & Schilman, 2018). Thus, survival will rely on the existence of genetic variation for increased heat tolerance present in natural populations (Bush et al., 2016).

The genus *Drosophila* consists of around two thousand species (Markow & O'Grady, 2006) and represents a vast collection of organisms adapted to a wide variety of environmental challenges. Some species, for example, have adapted to tropical environments with high temperatures over all seasons and others to temperate areas with seasonally cold climates characteristic of high latitudes or altitudes. While some studies show that mainly tropical species will be affected under future warming, as they are living close to their thermal‐safety margins (Deutsch et al., 2008), others highlighted that both tropical and widespread species will face a similar proportional reduction in their distribution range (Overgaard, Kearney, & Hoffmann, 2014). The case of *D. melanogaster* is intriguing because, although it has a tropical African origin, it exhibits a widespread cosmopolitan distribution. Although abundant genetic variation for heat tolerance in different natural populations of *D. melanogaster* has been shown (Fallis, Fanara, & Morgan, 2011; Sgrò et al., 2010), the identification of specific genes contributing to such variation is rarely reported.

In this work, we studied the genetic basis underlying tolerance to high temperatures in the fly *D. melanogaster*. In particular, we investigated whether this species harbors genetic variation that allows an expansion of its upper thermal limit through climatic adaptation. To do so, we characterized the phenotypic variation of CTmax in adult flies of 34 lines belonging to the *Drosophila* Genetic Reference Panel (DGRP), in order to perform a genomewide association study (GWAS) to screen for candidate genes that would potentially contribute to increased heat resistance in a warming scenario. Results are also discussed in relation of two populations from the sub‐Saharan ancestral range.

## 2 MATERIALS AND METHODS

### 2.1 *Drosophila melanogaster* stocks

We used a random sample of 34 homozygotic lines derived from a natural *D. melanogaster* population of Raleigh which belong to the DGRP (Huang et al., 2014) to measure the phenotypic response to high temperatures. Flies were reared on vials containing Carolina Formula 4‐24 Instant *Drosophila* Medium (Carolina Biological Supply Company, Burlington, NC) at 25°C with 12‐hr light/dark cycle.

### 2.2 Thermal tolerance measurements

Upper thermal tolerance was measured as CTmax using a dynamic method. Flies were individually placed on each of the 96 wells of an activity‐sensing device (Custom Minellidro, Sable Systems International (SSI), Las Vegas, NV) without cold or CO_2_ anesthesia. The activity device, which employed 96 low‐intensity, multiplexed 940 nm infrared light beams to detect activity via optical transmission variance, was placed inside a SSI PTC‐1 temperature control cabinet attached to a SSI PELT‐5 temperature controller. Each assay began with 15 min at 30°C after which temperature was programmed to increase at a rate of 0.25°C/min up to ca. 50°C. At this temperature, all flies reached their CTmax and their ULT. Chamber temperature was measured with a thermocouple attached to a SSI TC‐2000 thermocouple meter. The activity of each of the 96 flies was stored in a computer at 1 Hz by SSI ExpeData data acquisition software (v. 1.8.2). Phenotyping was conducted with randomly chosen lines measured simultaneously in randomly chosen wells (selected at random by a custom computer program with stochastic reseeding of the random number generator for each experiment) on each 96‐well array.

**Figure 1:**
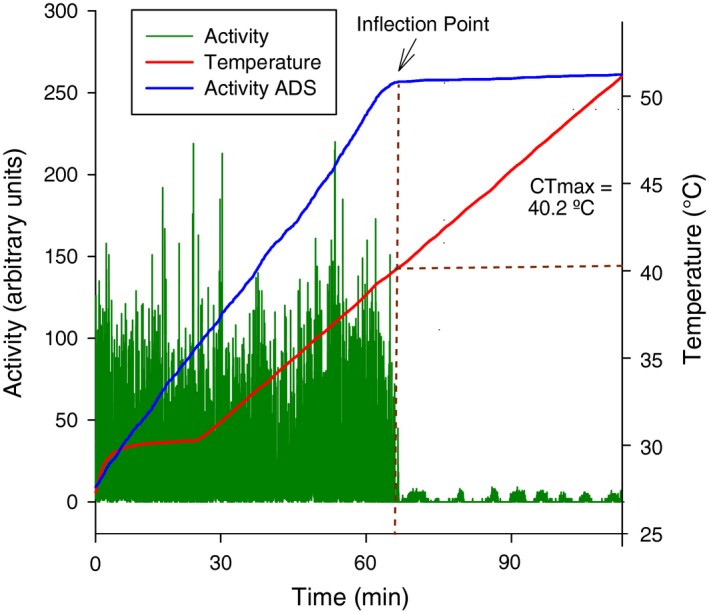
Example of an activity recording of an individual *Drosophila melanogaster* fly. Critical thermal maximum is obtained by extrapolation of absolute difference sum (ADS) inflection point to the temperature curve

Activity data were analyzed using SSI ExpeData data analysis software. CTmax was defined using the method proposed by Lighton and Turner (2004). In brief, activity, as measured by variance in optical transmission, was converted to the absolute difference sum (ADS), that is, the cumulative sum of the absolute difference between all adjacent data points. The inflection point in the ADS is suggested to be an objective method for identifying the point at which short‐term variability in the data declines abruptly, thus indicating the temperature at which insect ceases activity (Figure 1).

Thermal tolerance assays were performed on 1‐day‐old flies. Once each assay finished, we determined the sex of each individual.

All statistical analyses were performed using R version 3.3.1 (R Core Team, 2017). To analyze variation of CTmax across the measured strains, a maximum likelihood approach was applied for fitting a mixed effects model using *lmer* function in *lme4* package (Bates, Maechler, & Bolker, 2015). The model included sex as a fixed factor and line and its interaction with sex as random factors. The sex by line interaction was excluded from the model as it failed to increase the model goodness of fit. The significance of the random effects was assessed using the *rand* function in the *lmerTest* package (Kuznetsova, Brockhoff, & Christensen, 2017).

### 2.3 Genomewide association study for tolerance to high temperatures

The GWAS was performed on line means of 34 of the 205 DGRP lines (Huang et al., 2014) using the DGRP web tool (http://dgrp2.gnets.ncsu.edu). In brief, this analysis associates the phenotypic variation of CTmax with segregating single nucleotide polymorphisms (SNPs) present in the sampled DGRP lines. Thus, we can identify which regions of the genome (regulatory or coding) are associated with tolerance to high temperatures. Effects of SNPs were estimated as the average difference in trait mean between the major and minor alleles (the minor allele is the less frequent allele in the population). In addition, this analysis takes into account *Wolbachia* infection status as well as the major chromosomal inversions. For further details of GWAS, see supplementary materials of Mackay et al. (2012).

### 2.4 Comparison with sub‐Saharan populations

To quantify SNPs frequency in other populations, we downloaded genetic data from a Zambia population (Hervas, Sanz, Casillas, Pool, & Barbadilla, 2017), which would represent an ancestral range population (Pool et al., 2012). In addition, using the estimated percentage of African ancestry in the DGRP lines calculated by Pool (2015) using 27 genomes from Rwanda, we quantified which of the tested lines had a high probability of sub‐Saharan ancestry for each genomic region that contained a significant SNP.

### 2.5 Future climate change projections

To analyze the present and future incidence of extreme high temperatures on CTmax, we downloaded bias‐corrected raw data of climate layers from the CCAFS (Climate Change, Agriculture and Food Security)‐downscaled general circulation model (GCM) data portal (http://www.ccafs-climate.org/), in the form of data for one emissions scenario (RCP 6.0) and four GCMs: bcc‐csm1‐1, bcc‐csm1‐1‐m, CSIRO‐Mk3‐6‐0, and MIROC‐ESM. In particular, we downloaded maximum temperature data for Raleigh NC (Latitude = 35.763340, Longitude = −78.662644) from past periods (1980–2005) and future projections (2045–2070). For both periods, we used the ensemble data for the models.

## 3 RESULTS

**Figure 2:**
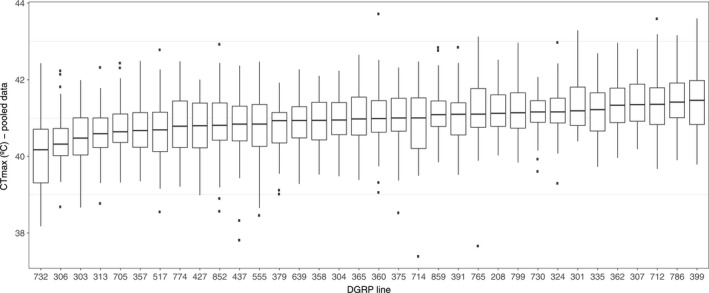
Critical thermal maxima of 34 *Drosophila* Genetic Reference Panel (DGRP) lines

**Figure 3:**
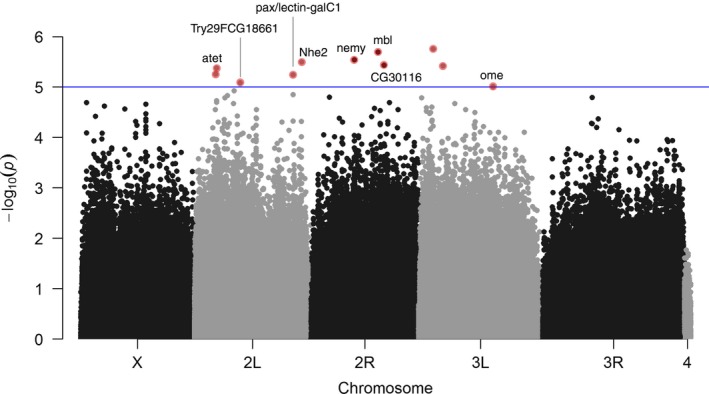
Manhattan plot for single nucleotide polymorphisms (SNPs) genomewide association study distribution. Each point represents a SNP. The height of the SNPs represents the strength of association with CTmax. Blue horizontal line represents the genomewide significance threshold (*p *=* *1 × 10^−5^)

A total of 1,837 flies were measured. Means of CTmax across DGRP lines ranged from 40.05 to 41.47°C, with a mean value of 40.98 ± 0.79 (*SD*) (Supporting Information Table S1 shows mean CTmax for each line and sex). In order to analyze genetic variation of CTmax between DGRP lines and sex, we performed a mixed effects model. Significant genetic variation across DGRP lines was found (*χ* ^2^ = 28.73; *p *=* *8 × 10^−8^), with a broad sense heritability (*H* ^2^) of 0.14. The effect of sex on CTmax was not significant (*t* = −1.44; *p *=* *0.16), nor its interaction with the line (*χ* ^2^ = 1.16; *p *=* *0.38). Thus, data from both sexes were pooled and GWAS performed on the mean CTmax value for each line (Figure 2). For the 34 lines measured, 1,288,487 SNPs were analyzed. The analysis showed no effect due to *Wolbachia* infection (ANOVA, *F*_1,3_ = 0.002, *p *=* *0.97). From the 16 identified large chromosomal inversions, 11 were monomorphic in the sample of used lines. One of the remaining inversions was significantly associated with CTmax (In_3R_K, ANOVA, *F*_1,3_ = 11.13, *p *=* *0.002); however, no SNPs were located within that region. At *p* < 10^−5^, there are 12 regions associated with CTmax (Figure 3 and Supporting Information Table S2). While four SNPs are located within intergenic regions, the other eight SNPs mapped within genes.

**Figure 4:**
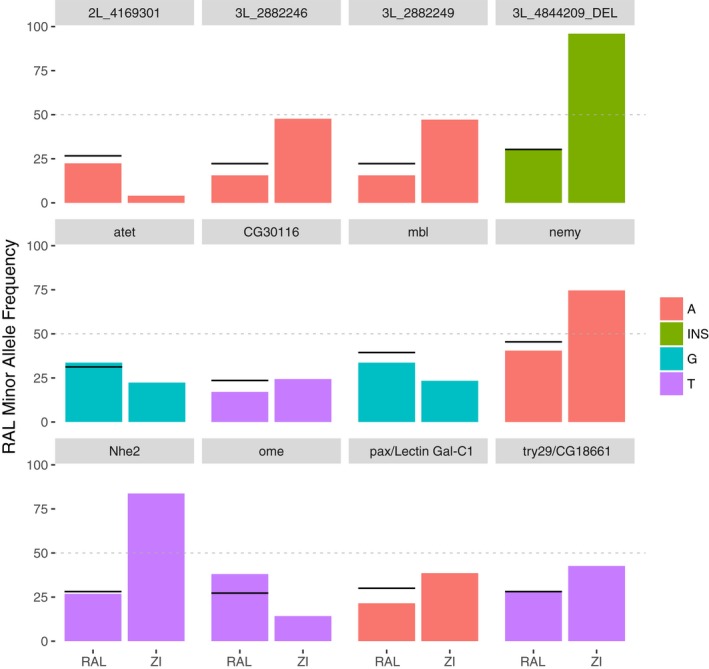
Alleles’ frequencies from Raleigh (RAL) and Zambia (ZI) populations. Minor alleles from the significant single nucleotide polymorphisms (SNPs) associated with CTmax and their frequency in 205 *Drosophila* Genetic Reference Panel (DGRP) lines from RAL and frequency in 197 lines from ZI. Black line indicates the allele frequency of the 34 DGRP lines used in this study. Gene ID for each SNP is written over bar plot. Dotted gray line indicates 50% frequency. Bar colors correspond to the minor allele found in RAL. Colors refer to minor alleles from the RAL population; in particular, INS denotes the insertion sequence: CAGGGTATACAG

**Figure 5:**
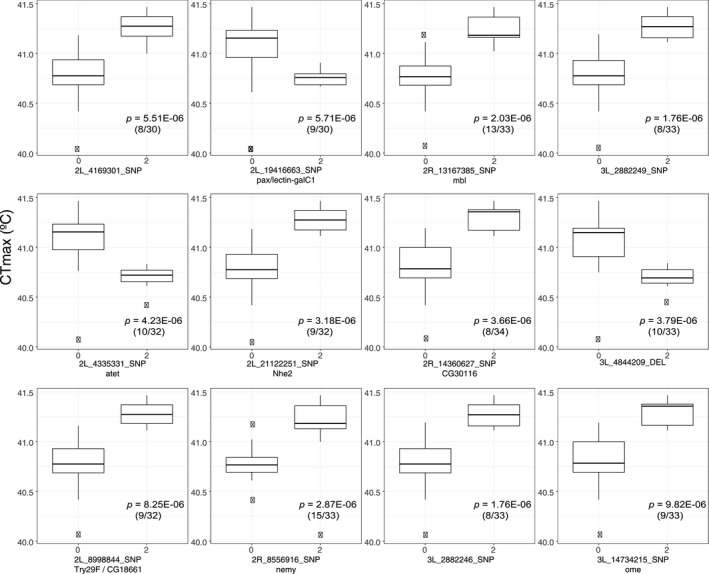
Box plots of the significant single nucleotide polymorphisms (SNPs) associations in the genomewide association study. For each SNP, the lines are partitioned into two groups: lines containing the major (0) or the minor allele (2). The *y*‐axis shows CTmax values. Box plots are ordered by significance level, and below each *p*‐value, the proportion of lines which contain the minor allele variant is noted between brackets

Within our sample, minor allele frequency ranged from 22.2% to 45.5%, which is similar to the frequencies found for the SNPs of the whole DGRP lines (see left columns of Figure 4 and Supporting Information Table S3). In addition, major and minor alleles of these SNPs are also found in a sub‐Saharan population (Zambia) (Supporting Information Table S3 and Figure 4). In most of the SNPs associated with CTmax (9 of 12), the minor alleles increased the upper thermal limit (Figure 5). On average, lines containing the minor allele that increased CTmax values raised heat tolerance by 0.38 ± 0.04°C. Most of them (7 of 9) remained as minor alleles in Zambia (Africa) population. However, the less frequent alleles for *nemy* and *Nhe2* in Raleigh population are mayor alleles in Africa population (Figure 4).

## 4 DISCUSSION

In this study, we measured critical thermal maxima (CTmax) in a subset of lines of the DGRP with a new activity‐sensing device and performed a GWAS. Through this analysis, we obtained 12 novel significant SNPs along the genome that are associated with CTmax. In most of these SNPs, the minor alleles increased the upper thermal limit suggesting that this natural population harbors raw genetic variation for expanding its heat tolerance in the context of the future climate warming. DGRP lines are derived from females inseminated in the wild in a single natural population of *D. melanogaster*, which after several generations of full‐sibling inbreeding in the laboratory reached the high level of homozygosity necessary for GWAS (Mackay et al., 2012). However, the ancestry of these flies can be traced to European and sub‐Saharan populations (Pool, 2015), suggesting that their genetic background is representative of natural variations. The ancestry varies along the chromosomes and the different DGRP lines (Pool, 2015). Here, 11 of 12 significant SNPs mapped within regions that have African ancestry (Supporting Information Figure S1). Thus, despite inbreeding, our results illustrate sufficient natural genetic variation, and therefore adaptive potential, for elevating CTmax via natural selection.

Increases in temperature will affect the normal functioning of cells and organisms by loss of protein structure and stability, membrane collapse, disruption of internal organization of cells, and failure in neural activity (Angilletta, 2009; Richter, Haslbeck, & Buchner, 2010). Richter et al. (2010) reviewed which genes were induced in the heat shock response from various species and studies on a genomewide scale. They grouped their results in 7 classes of proteins involved in this response; metabolism, DNA/RNA repair, molecular chaperones, cell organization, transport and detoxification, and protein degradation. Here, we found a protein related to cell organization: *paxillin (pax)* is a cytoskeletal scaffolding protein. This protein is also associated with macromolecule recycling because it has a critical role in autophagosome formation (Chen et al., 2008). Also, there are two protein‐coding genes associated with transport through plasma membranes among the significant SNPs, that is, *atet*, and *Nhe2*. The latter is also associated with ion and pH homeostasis being a Na^+^: H^+^ exchanger (Giannakou & Dow, 2001). Because protein stability is not only affected by temperature but also by other factors such as pH (Hochachka & Somero, 2002), these ion channels might play a role in maintaining protein stability as temperature rises. Another important effect of heat shock is the failure of neural activity, particularly in axonal conduction and synaptic transmission (Robertson, 2004; Robertson & Money, 2012). One of the candidate genes identified here is *nemy* which has been previously associated with neurotransmitter release (Iliadi et al., 2008; Knight et al., 2015).

None of the heat shock protein genes (*hsp*) were among the candidate genes resulting from the GWAS in the present study. These molecular chaperones are highly expressed during and after heat exposure (Birch‐Machin et al., 2005; Jensen, Nielsen, & Loeschcke, 2008; Sørensen, Nielsen, Kruhøffer, Justesen, & Loeschcke, 2005). However, our study did not involve thermal acclimation. In addition, because of the short duration of the assay (less than 55 min) as a consequence of temperature ramping rate and based on previous results (Sørensen, Loeschcke, & Kristensen, 2013), we assume that *hsps* expression would have not been achieved. Indeed, it would be interesting to test in the future if *hsps* show polymorphic variants in response to short‐ and long‐term heat acclimation and slower ramping rates that allow for hardening effects. Moreover, it has been previously proposed that there are high costs of maintaining alleles which lead to elevated basal expression of *hsps* (Sørensen, Kristensen, & Loeschcke, 2003) and consequently adaptation to higher temperatures might be driven by other genes.

The majority of the SNPs in our sample are located within intronic regions, suggesting that variation in heat tolerance can be mediated by changes in gene expression, as some introns harbor regulatory elements. For example, there are 5 SNPs that map to regions with putative transcription factor binding sites (*atet*,* pax*, and *Try29F‐CG18661*, 3L2882246, and 3L2882249). In this line, one SNP which harbors two candidate genes appears to be associated with heat tolerance; *lectin‐galC1* has higher expression in tropical populations, and its expression varies in response to environmental temperature (Juneja, Quinn, & Jiggins, 2016; Levine, Eckert, & Begun, 2011; Zhao, Wit, Svetec, & Begun, 2015), while *Pax* is upregulated in flies selected for heat tolerance (Nielsen, Sørensen, Kruhøffer, Justesen, & Loeschcke, 2006).

In order to determine whether CTmax is associated with other traits related to thermal tolerance, we performed correlations between our data and other traits measured in DGRP lines. The correlation between CTmax and chill coma recovery time (Mackay et al., 2012) do not show significant association (*t* = −0.81, *p *=* *0.42, *r* = −0.10). CTmax and time to heat knockdown (Duun Rohde et al., 2016) were carried out using data from females, as the latter was measured on ~5‐day‐old females. We found no correlation between CTmax and time to heat knockdown (*t* = 0.45, *p *=* *0.66, *r* = 0.09). An important factor that could explain the lack of correlation between these measurements is age difference between tested flies. Heat tolerance is age‐dependent (Pappas, Hyde, Bowler, Loeschcke, & Sørensen, 2007), and here, we chose to use 1‐day‐old flies to be certain that flies have not mated, while Duun Rohde et al. (2016) used 5‐day‐old flies. In addition, there is contrasting evidence on the correlation between different heat tolerance measurements. While an artificial selection study shows that selection for static heat knockdown time resulted in increased tolerance to ramping assays (Hangartner & Hoffmann, 2016) suggesting a common mechanism involved in heat tolerance, others show a lack of correlation among these metrics (Blackburn, van Heerwaarden, Kellermann, & Sgrò, 2014; Hoffmann, Dagher, Hercus, & Berrigan, 1997).

The method used to measure upper thermal limits has been the subject of multiple analyses and discussions (Overgaard, Kristensen, & Sørensen, 2012; Rezende, Tejedo, & Santos, 2011; Terblanche et al., 2011). Heat tolerance estimates obtained from static methods differ from those obtained by dynamic (i.e., ramping) ones. Through theoretical approaches, it has been argued that dynamic methods add confounding effects such as dehydration and resource depletion (Rezende et al., 2011; Santos, Castañeda, & Rezende, 2011). However, other papers evaluate empirically the theoretical predictions proposed by these models, concluding that slow ramping assays are more ecologically relevant (Overgaard et al., 2012; Terblanche et al., 2011). Our CTmax values were similar to those obtained in previous studies with the same rate of temperature increase (Chown, Jumbam, Sørensen, & Terblanche, 2009; Hangartner & Hoffmann, 2016). Heritability estimates of heat tolerance tend to be negligible in slow ramping assays (Blackburn et al., 2014; Mitchell & Hoffmann, 2010). Our findings, however, show that there is, albeit low, heritable variation for CTmax in ramping assays.

**Figure 6:**
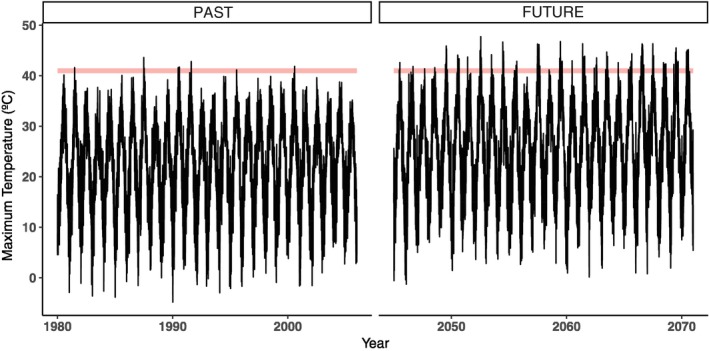
Relationship between maximum temperature and thermal limits for *Drosophila melanogaster* from Raleigh NC. Maximum temperature is depicted in black lines for the current period (1980–2005) and future projection (2045–2070). Light red bars are the critical thermal maximum (CTmax) range (40.5–41.47°C). Extreme temperatures from 1980 to 2005 show low proportion of days above CTmax (10 days), and future climate scenario used (RCP 6.0) shows 243 for CTmax

Besides finding heritable variation of CTmax in this subset of DGRP flies, another key finding is that, in most of the SNPs associated with this character, the minor alleles (within the measured lines) increased the upper thermal limit by a mean value of 0.38°C. This suggests that this natural population harbors raw genetic variation for expanding its heat tolerance. Similar adaptive potential has been recorded in artificial selection experiments with an increase in heat tolerance of 0.5°C (Hangartner & Hoffmann, 2016). Climatic records show a very low incidence of days above CTmax temperature (10 days) from 1980 to 2005 in Raleigh. Over the coming decades (2045–2070), however, future climate scenarios predict an increase in the number of days (243 days) with extreme high temperature above CTmax (Figure 6). In addition, preliminary data for this species show that ULT is very close to its CTmax, that is, 1.35°C above (data not shown). Thus, predicted rising temperatures might drive the evolution of heat tolerance in this natural population to some extent. A sub‐Saharan ancestral range population (Zambia), where mean and maximum temperature are higher than in Raleigh (Supporting Information Table S4), possesses the 12 SNPs that we found to be associated with CTmax in the DGRP. Two of these minor alleles that increase CTmax (associated with *Nhe2* and *nemy*) are major alleles in the population from Zambia (Figure 4), which indirectly support the involvement of these candidate genes in increasing CTmax. Further testing in this *D. melanogaster* population as well as others from different latitudes or other species will help to understand the generalization of this pattern. To this end, here we provide candidate loci and SNPs to be tested in future studies aiming to understand the impact of climate warming on insect species evolution.

## CONFLICT OF INTEREST

None declared.

## AUTHOR CONTRIBUTIONS

CR, JRBL, PES, and JM conceived and designed the study. CR performed the experiments. GV performed the climatic analysis. CR and JM analyzed the data and wrote the first draft of the manuscript. All authors wrote and approved the final version of the manuscript.

## DATA ACCESSIBILITY

Data are available from the FigShare Digital Repository: https://doi.org/10.6084/m9.figshare.6741668.
