# Supplementary figures and images for: Genetic variation for tolerance to high temperatures in a population of Drosophila melanogaster
Source: Ecol Evol. 2018 Oct 11;8(21):10374–83. doi: 10.1002/ece3.4409 (PMC6238130; doi:10.1002/ece3.4409)

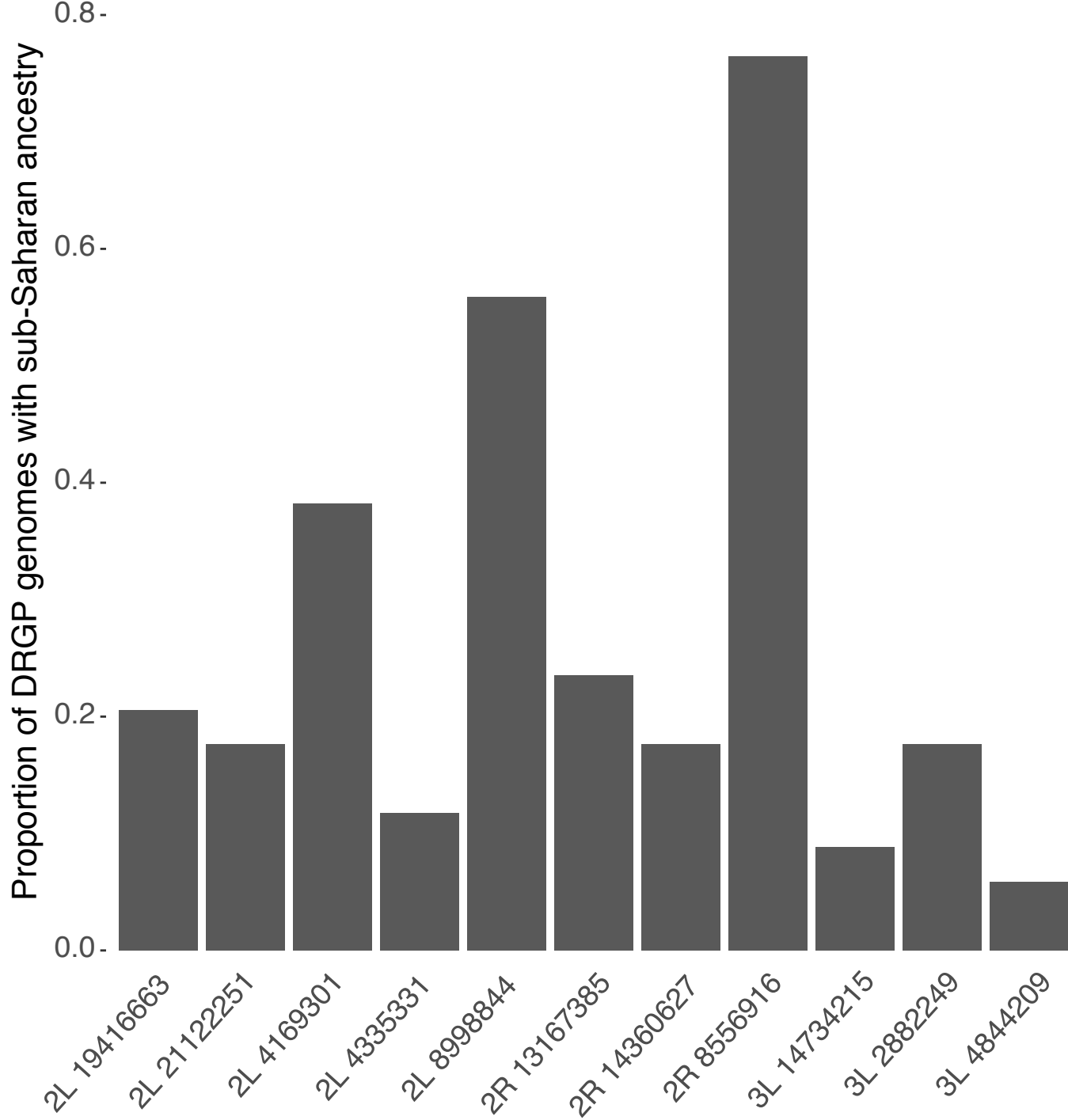

Supplement: Supplementary file 1 [file ECE3-8-10374-s001.pdf]
