# Supplementary material for: Genetic variation for tolerance to high temperatures in a population of Drosophila melanogaster
Source: Ecol Evol. 2018 Oct 11;8(21):10374–83. doi: 10.1002/ece3.4409 (PMC6238130; doi:10.1002/ece3.4409)
Supplement: Supplementary file 2 [file ECE3-8-10374-s002.docx]

| DGRP ID | sex | N | mean | se |
| --- | --- | --- | --- | --- |
| 208 | male | 28 | 41.25 | 0.12 |
|  | female | 23 | 41.11 | 0.13 |
| 301 | male | 23 | 41.41 | 0.15 |
|  | female | 35 | 41.26 | 0.10 |
| 303 | male | 23 | 40.59 | 0.17 |
|  | female | 15 | 40.40 | 0.23 |
| 304 | male | 29 | 40.99 | 0.12 |
|  | female | 20 | 41.01 | 0.15 |
| 306 | male | 20 | 40.71 | 0.18 |
|  | female | 26 | 40.20 | 0.12 |
| 307 | male | 37 | 41.25 | 0.09 |
|  | female | 31 | 41.55 | 0.13 |
| 313 | male | 29 | 40.67 | 0.15 |
|  | female | 31 | 40.55 | 0.09 |
| 324 | male | 20 | 41.31 | 0.17 |
|  | female | 24 | 41.09 | 0.14 |
| 335 | male | 18 | 41.11 | 0.19 |
|  | female | 16 | 41.22 | 0.16 |
| 357 | male | 30 | 40.68 | 0.13 |
|  | female | 28 | 40.66 | 0.12 |
| 358 | male | 31 | 41.05 | 0.11 |
|  | female | 23 | 40.76 | 0.13 |
| 360 | male | 30 | 40.90 | 0.10 |
|  | female | 28 | 41.24 | 0.18 |
| 362 | male | 30 | 41.42 | 0.13 |
|  | female | 46 | 41.31 | 0.09 |
| 365 | male | 29 | 41.07 | 0.11 |
|  | female | 24 | 41.01 | 0.18 |
| 375 | male | 38 | 41.05 | 0.11 |
|  | female | 23 | 40.93 | 0.18 |
| 379 | male | 21 | 40.81 | 0.14 |
|  | female | 21 | 40.72 | 0.16 |
| 391 | male | 32 | 41.03 | 0.12 |
|  | female | 34 | 41.02 | 0.12 |
| 399 | male | 28 | 41.70 | 0.17 |
|  | female | 24 | 41.19 | 0.19 |
| 427 | male | 28 | 40.64 | 0.14 |
|  | female | 35 | 40.90 | 0.11 |
| 437 | male | 34 | 40.93 | 0.12 |
|  | female | 26 | 40.84 | 0.15 |
| 517 | male | 34 | 40.53 | 0.12 |
|  | female | 27 | 40.90 | 0.16 |
| 555 | male | 23 | 40.74 | 0.20 |
|  | female | 28 | 40.79 | 0.15 |
| 639 | male | 39 | 40.89 | 0.11 |
|  | female | 27 | 40.93 | 0.14 |
| 705 | male | 28 | 40.75 | 0.10 |
|  | female | 26 | 40.75 | 0.15 |
| 712 | male | 21 | 41.38 | 0.19 |
|  | female | 36 | 41.35 | 0.11 |
| 714 | male | 18 | 41.03 | 0.16 |
|  | female | 19 | 40.86 | 0.21 |
| 730 | male | 25 | 41.21 | 0.08 |
|  | female | 22 | 41.01 | 0.13 |
| 732 | male | 20 | 40.33 | 0.18 |
|  | female | 27 | 39.83 | 0.20 |
| 765 | male | 25 | 41.24 | 0.13 |
|  | female | 26 | 41.20 | 0.14 |
| 774 | male | 27 | 40.68 | 0.15 |
|  | female | 25 | 40.91 | 0.16 |
| 786 | male | 26 | 41.38 | 0.14 |
|  | female | 27 | 41.49 | 0.13 |
| 799 | male | 29 | 41.27 | 0.12 |
|  | female | 24 | 41.07 | 0.16 |
| 852 | male | 33 | 40.98 | 0.13 |
|  | female | 28 | 40.66 | 0.18 |
| 859 | male | 24 | 41.22 | 0.15 |
|  | female | 29 | 41.11 | 0.10 |

**Table S1.** CTmax for 37 DGRP lines. Means and standard errors for all lines measured.
