# Supplementary material for: Genetic variation for tolerance to high temperatures in a population of Drosophila melanogaster
Source: Ecol Evol. 2018 Oct 11;8(21):10374–83. doi: 10.1002/ece3.4409 (PMC6238130; doi:10.1002/ece3.4409)
Supplement: Supplementary file 3 [file ECE3-8-10374-s003.docx]

| ID | Minor  Allele | Major  Allele | Ref  Allele | Mixed model P-val | Flybase ID | Gene ID | Genomic annotation | Function  (Selected) | Max expression  (fly atlas) |
| --- | --- | --- | --- | --- | --- | --- | --- | --- | --- |
| 3L_2882246_SNP | A | C | C | 1.76E-06 | NA | NA |  |  |  |
| 3L_2882249_SNP | A | T | T | 1.76E-06 | NA | NA |  |  |  |
| 2R_13167385_SNP | G | A | A | 2.03E-06 |  | mbl | FBgn0265487 | RNA binding protein, regulator of alternative splicing | Larval fat body |
| 2R_8556916_SNP | A | C | A | 2.87E-06 | FBgn0261673 | Nemy | INTRON | carbon-monoxide oxygenase activity  locomotor activity  regulation of neurotransmitter secretion | Tubule and hindgut |
| 2L_21122251_SNP | T | C | C | 3.18E-06 | FBgn0040297 | Nhe2 | DOWNSTREAM  SYNONYMOUS_CODING | ion and pH homeostasis | Larval hindgut |
| 2R_14360627_SNP | T | A | A | 3.66E-06 | FBgn0028496 | CG30116 | INTRON |  | Crop and brain |
| 3L_4844209_DEL | CAGGGTATACAG | TC | CAGGGTATACAG | 3.79E-06 | NA | NA |  |  |  |
| 2L_4335331_SNP | G | C | C | 6.89E-07 | FBgn0020762 | atet | INTRON | Transmembrane transporter activity | Eye |
| 2L_4169301_SNP | A | G | G | 5.51E-06 | NA | NA |  |  |  |
| 2L_19416663_SNP | A | T | T | 5.71E-06 | FBgn0041789 | Pax | INTRON | Adaptor protein associated with integrins  Cell homeostasis- autophagosome formation | S2 cells (growing) |
| 2L_19416663_SNP | A | T | T | 5.71E-06 | FBgn0016675 | Lectin-galC1 | UPSTREAM | Receptor | Heart |
| 2L_8998844_SNP | T | A | A | 2.53E-05 | FBgn0015316 | Try29F | SYNONIMOUS CODING | Protein metabolism | Midgut |
| 2L_8998844_SNP | T | A | A | 2.53E-05 | FBgn0040964 | CG18661 | DOWNSTREAM |  | Thoracicoabdominal ganglion |
| 3L_14734215_SNP | T | A | T | 9.82E-06 | FBgn0259175 | ome | INTRON | Protein metabolism- serine protease | Midgut and salivary gland |

**Table S2-** Top GWAS results for CTmax. Blue rows correspond to SNPs located in intergenic regions. Green rows correspond to one SNP that is shared by two genes.
