# Supplementary material for: Genetic variation for tolerance to high temperatures in a population of Drosophila melanogaster
Source: Ecol Evol. 2018 Oct 11;8(21):10374–83. doi: 10.1002/ece3.4409 (PMC6238130; doi:10.1002/ece3.4409)
Supplement: Supplementary file 4 [file ECE3-8-10374-s004.docx]

| id | Gene id | Minor allele freeze 2 | Major allele freeze 2 | Minor allele freq freeze 2 (%) | Major allele freq freeze 2 (%) | Minor allele sample | Major allele sample | Minor allele freq sample (%) | Minor allele freq zambia | Major allele freq zambia |
| --- | --- | --- | --- | --- | --- | --- | --- | --- | --- | --- |
| 3L_2882246_SNP | NA | A | C | 15.610 | 81.951 | A | C | 22.242 | A 0.4771574 | C 0.5228426 |
| 3L_2882249_SNP | NA | A | T | 15.122 | 82.439 | A | T | 22.242 | A 0.4720812 | T 0.5279188 |
| 2R_13167385_SNP | mbl | G | A | 33.171 | 62.439 | G | A | 39.394 | G 0.2335025 | A  0.7664975 |
| 2R_8556916_SNP | Nemy | A | C | 40.976 | 53.659 | A | C | 45.454 | **C 0.2538071** | **A 0.7461929** |
| 2L_21122251_SNP | Nhe2 | T | C | 33.200 | 58.537 | T | C | 28.125 | **C 0.1624365** | **T 0.8375635** |
| 2R_14360627_SNP | CG30116 | T | A | 16.098 | 82.927 | T | A | 23.529 | T 0.2436548 | A 0.7563452 |
| 3L_4844209_DEL | NA | CAGGGTATACAG | TC | 30.244 | 65.366 | CAGGGTATACAG | TC | 30.303 | **TC**  **0.04060914** | **INS**  **0.9593909** |
| 2L_4335331_SNP | atet | G | C | 34.146 | 58.049 | G | C | 31.250 | G 0.2233503 | C 0.7766497 |
| 2L_4169301_SNP | NA | A | G | 20.000 | 73.171 | A | G | 26.667 | A 0.04060914 | G 0.94923858 |
| 2L_19416663_SNP | Pax/ Lectin-galC1 | A | T | 22.439 | 66.829 | A | T | 30 | A 0.385786802 | T 0.609137056 |
| 2L_8998844_SNP | CG18661/ Try29F | T | A | 28.780 | 64.787 | T | A | 28.125 | T 0.4263959 | A 0.5736041 |
| 3L_14734215_SNP | ome | T | A | 39.024 | 56.098 | T | A | 27.273 | T 0.142132 | A 0.857868 |

**Table S3-** Allele frequencies for significant SNPs in 205 DGRP genomes (Freeze 2), the 34 DGRP samples used in this study and 197 ancestral range population genomes from Zambia (ZI).
