# Supplementary material for: Genetic variation for tolerance to high temperatures in a population of Drosophila melanogaster
Source: Ecol Evol. 2018 Oct 11;8(21):10374–83. doi: 10.1002/ece3.4409 (PMC6238130; doi:10.1002/ece3.4409)
Supplement: Supplementary file 5 [file ECE3-8-10374-s005.docx]

| Population Sample | Latitude | Altitude (m) | Mean Tmax (°C) | S.D. | Max Tmax (°C) |
| --- | --- | --- | --- | --- | --- |
| Zambia, Siavonga (ZI) | -16.50 | 530 | 31.58 | 3.15 | 44.41 |
| USA, Raleigh (RAL) | -78.67 | 91 | 22.01 | 7.75 | 38.26 |

**Table S4-** *D. melanogaster* inbred lines from Raleigh and Zambia and environmental variables.
